# Genome Sequence of a Tigecycline-Resistant Clinical Isolate of *Acinetobacter baumannii* Strain AB031 Obtained from a Bloodstream Infection

**DOI:** 10.1128/genomeA.01036-14

**Published:** 2014-10-16

**Authors:** Peter C. Loewen, Yasser Alsaadi, Dinesh Fernando, Ayush Kumar

**Affiliations:** a Department of Microbiology, University of Manitoba, Winnipeg, Manitoba, Canada; b Department of Medical Microbiology, University of Manitoba, Winnipeg, Manitoba, Canada

## Abstract

We report here the 3.8-Mbp genome sequence of a blood isolate of *Acinetobacter baumannii* strain AB031.

## GENOME ANNOUNCEMENT

*Acinetobacter baumannii* is a Gram-negative bacterium that is notorious for causing infections that can be extremely difficult to treat because of its high resistance to all of the antibiotics currently in clinical use (1, 2). As a result of its high resistance, *A. baumannii* is considered a significant threat to human health, particularly to individuals with compromised immunity.

*A. baumannii* strain AB031 was isolated from a bloodstream infection in a 55-year-old female patient. It displays resistance to tigecycline (MIC, 8 µg/mL) and trimethoprim/sulfamethoxazole (MIC, 4 µg/mL) (3). However, it is susceptible to aminoglycosides (amikacin, gentamicin); carbapenems (imipenem, meropenem); fluoroquinolones (ciprofloxacin, levofloxacin, moxifloxacin); penicillin and β-lactamase inhibitors (tazobactam-piperacillin); and extended-spectrum cephalosporins (cefepime, ceftriaxone) (3). We have shown previously that *A. baumannii* AB031 displays overexpression of the AdeABC efflux pump (3). This can therefore at least partly explain the tigecycline resistance of *A. baumannii* AB031, because the AdeABC pump has been implicated in tigecycline resistance of clinical isolates of *A. baumannii* (4). In addition, it also displays in approximately three times more biofilm formation compared to the type strain *A. baumannii* ATCC19606 and shows surface-associated motility (Y. Alsaadi, D. Fernando, and A. Kumar, unpublished data). The factors contributing to the enhanced resistance of *A. baumannii* AB031 to tigecycline and its ability to form biofilms will be published elsewhere.

DNA was isolated using the Ultra-Clean Microbial DNA isolation kit (MoBio Laboratories, Carlsbad, CA, USA) following the manufacturer’s instructions. Genome sequencing was performed using the PacBio platform at the Genome Quebec facility (Montreal, QC, Canada). Sequencing was carried out using three SMRTcells, and combining the data provided a total of 88.9× coverage to give a single contiguous genome sequence. Assembly of the sequence was done using the PacBio SMRT Analysis pipeline version 2.2.0. The sequence was annotated by the National Center for Biotechnology Information (NCBI) Prokaryotic Genomes Annotation Pipeline.

The genome consists of 3,803,317 bases with a G+C content of 39.2%. There are a total of 3,564 putative genes, which include 3,456 protein-, 18 rRNA-, and 73 tRNA-coding sequences.

### Nucleotide sequence accession number.

The genome sequence of *A. baumannii* AB031 was deposited in NCBI GenBank under the accession number CP009256.
